# Photocatalytic Self-Fenton System of g-C_3_N_4_-Based for Degradation of Emerging Contaminants: A Review of Advances and Prospects

**DOI:** 10.3390/molecules28155916

**Published:** 2023-08-06

**Authors:** Zhouze Chen, Yujie Yan, Changyu Lu, Xue Lin, Zhijing Fu, Weilong Shi, Feng Guo

**Affiliations:** 1 School of Material Science and Engineering, Jiangsu University of Science and Technology, Zhenjiang 212003, Chinayyj1575240510@163.com (Y.Y.); 2 School of Water Resource and Environment, Hebei Province Key Laboratory of Sustained Utilization and Development of Water Recourse, Hebei Geo University, Shijiazhuang 050031, China; 3 School of Material Science and Engineering, Beihua University, Jilin 132013, China; 4 School of Energy and Power, Jiangsu University of Science and Technology, Zhenjiang 212003, China

**Keywords:** emerging pollutants, photocatalytic self-Fenton system, g-C_3_N_4_, modification strategy, environmental remediation

## Abstract

The discharge of emerging pollutants in the industrial process poses a severe threat to the ecological environment and human health. Photocatalytic self-Fenton technology combines the advantages of photocatalysis and Fenton oxidation technology through the in situ generation of hydrogen peroxide (H_2_O_2_) and interaction with iron (Fe) ions to generate a large number of strong reactive oxygen species (ROS) to effectively degrade pollutants in the environment. Graphite carbon nitride (g-C_3_N_4_) is considered as the most potential photocatalytic oxygen reduction reaction (ORR) photocatalyst for H_2_O_2_ production due to its excellent chemical/thermal stability, unique electronic structure, easy manufacturing, and moderate band gap (2.70 eV). Hence, in this review, we briefly introduce the advantages of the photocatalytic self-Fenton and its degradation mechanisms. In addition, the modification strategy of the g-C_3_N_4_-based photocatalytic self-Fenton system and related applications in environmental remediation are fully discussed and summarized in detail. Finally, the prospects and challenges of the g-C_3_N_4_-based photocatalytic self-Fenton system are discussed. We believe that this review can promote the construction of novel and efficient photocatalytic self-Fenton systems as well as further application in environmental remediation and other research fields.

## 1. Introduction

Since the middle of the 20th century, the rapid development of industrialization has appeared together with increasingly serious environmental problems, and the continuous release of toxic substances has led to the discovery of more and more emerging pollutants (e.g., antibiotics, dyes, etc.) in surface water, resulting in serious environmental pollution problems [1,2,3,4,5]. An increasing number of pollutants such as industrial dyes, antibiotics and agricultural chemicals exist widely in very low concentrations in the environment and thus are difficult to biodegrade, so the effective removal of these toxic species is related to human and biological health as well as sustainable reproduction [6,7,8].

In response to these problems, scientists have developed a series of water pollution treatment methods such as adsorption, flocculation, photodegradation, water splitting, and advanced oxidation processes (AOPs) [9,10,11,12,13,14,15]. Among them, AOPs are regarded as the most potential green treatment process to solve the problem of water pollution due to its strong oxidation capacity and without selectivity in the oxidation process, which has attracted global attention [16,17]. However, the traditional photocatalytic degradation technology relies on the active species produced by the semiconductor catalyst under light irradiation, which leads to a slow reaction rate, limited reaction applicability, and high light dependence, thus affecting its degradation performance. It is worth noting that photo-Fenton technology is considered to be one of the most cost-effective AOPs due to its nature of using sufficient ferrous ions (Fe^2+^) to activate hydrogen peroxide (H_2_O_2_) reactions to generate hydroxyl radicals (•OH) with strong oxidation capacity and sufficiently degrade organic pollutants to eventually mineralize into harmless small molecules (carbon dioxide, water, etc.), thus having important application potential in fields such as environmental remediation and waste treatment [18,19,20,21,22]. However, the single Fenton technology greatly limits its application in the field of degraded organic pollutants due to the following defects: (i) a large amount of H_2_O_2_ is added, resulting in a high processing cost [23,24]; (ii) the slower reduction kinetics of Fe^3+^/Fe^2+^ hinders the reaction efficiency and fails to adequately mineralize organic pollutants [25,26,27,28,29]. From the perspective of sustainable development, the photocatalytic self-Fenton system can organically combine photocatalytic with Fenton oxidation technology, which can not only solve the risk problem of adding extra H_2_O_2_ in the traditional Fenton technology, but can also accelerate the Fe^2+^/Fe^3+^ cycle, thus improving the removal efficiency of emerging pollutants [30]. Photocatalytic self-Fenton technology is mainly through the use of sunlight, which makes the photocatalyst, through photocatalytic oxygen reduction reaction (ORR) in situ, generate hydrogen peroxide (H_2_O_2_), and promotes the occurrence of heterogeneous self-Fenton reactions through the chain reaction with Fe^2+^ to generate strong oxidizing radical active species (e.g., •OH, $O_{2}^{-}$, ^1^O_2_), thus degrading and mineralizing organic pollutants in the water environment to form environmentally friendly species [31,32,33].

In recent years, researchers have made unremitting efforts to develop a series of photocatalytic materials with efficient H_2_O_2_ production such as inorganic semiconductors like TiO_2_ [34], CdS [35], graphite carbon nitride (g-C_3_N_4_) [36,37,38], and triazine covalent organic framework (CTF) [39]. Among them, g-C_3_N_4_, as a new organic semiconductor material, is a potential ORR photocatalyst due to its unique melamine (melam) structure, which is conducive to the reduction of O_2_ molecules to H_2_O_2_ through the two-electron path [40,41,42,43,44]. In addition, g-C_3_N_4_ has the advantages of high stability, easy preparation, low cost, and non-toxicity, and has attracted widespread attention in the field of photocatalysis since it was first applied by Wang et al. [45]. For example, Ma et al. used doped phosphorus (P) atomic modified g-C_3_N_4_ to promote the separation of photon-generated carriers, which increased the yield of in situ generated H_2_O_2_, and added FeSO_4_·7H_2_O as the source of Fe^2+^ in the system to promote the degradation effect of photocatalytic-self-Fenton technology on organic pollutants [46].

**Scheme 1:**
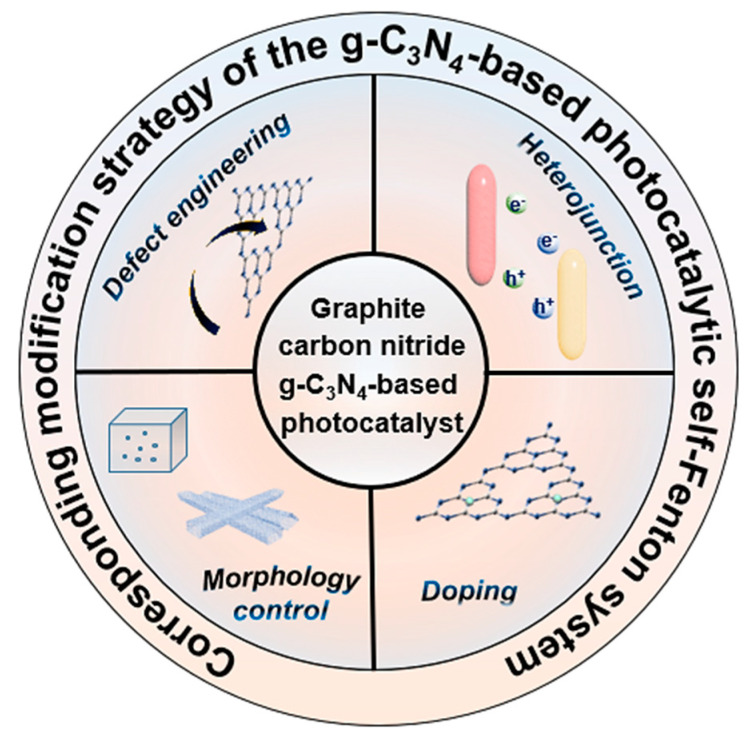
Modification strategies for the g-C_3_N_4_-based photocatalytic self-Fenton system are briefly described.

However, the existing reviews have not provided a complete and detailed overview of g-C_3_N_4_-based photocatalytic self-Fenton systems. Therefore, this review summarizes the research progress of a g-C_3_N_4_-based photocatalysis self-Fenton system. In the following sections, we will start with the photocatalysis self-Fenton reaction mechanism and comprehensively summarize the modification methods of g-C_3_N_4_-based photocatalysis self-Fenton technology for the efficient degradation of emerging pollutants (Scheme 1) including heterojunction, element doping, morphology regulation, defect engineering, and other modification methods. At length, the prospects and challenges of the photocatalytic self-Fenton system are discussed from the perspective of high efficiency, green, and safety.

## 2. Fundamental Mechanisms of Degradation of Pollutants by Photocatalysis Self-Fenton Technology

**Scheme 2:**
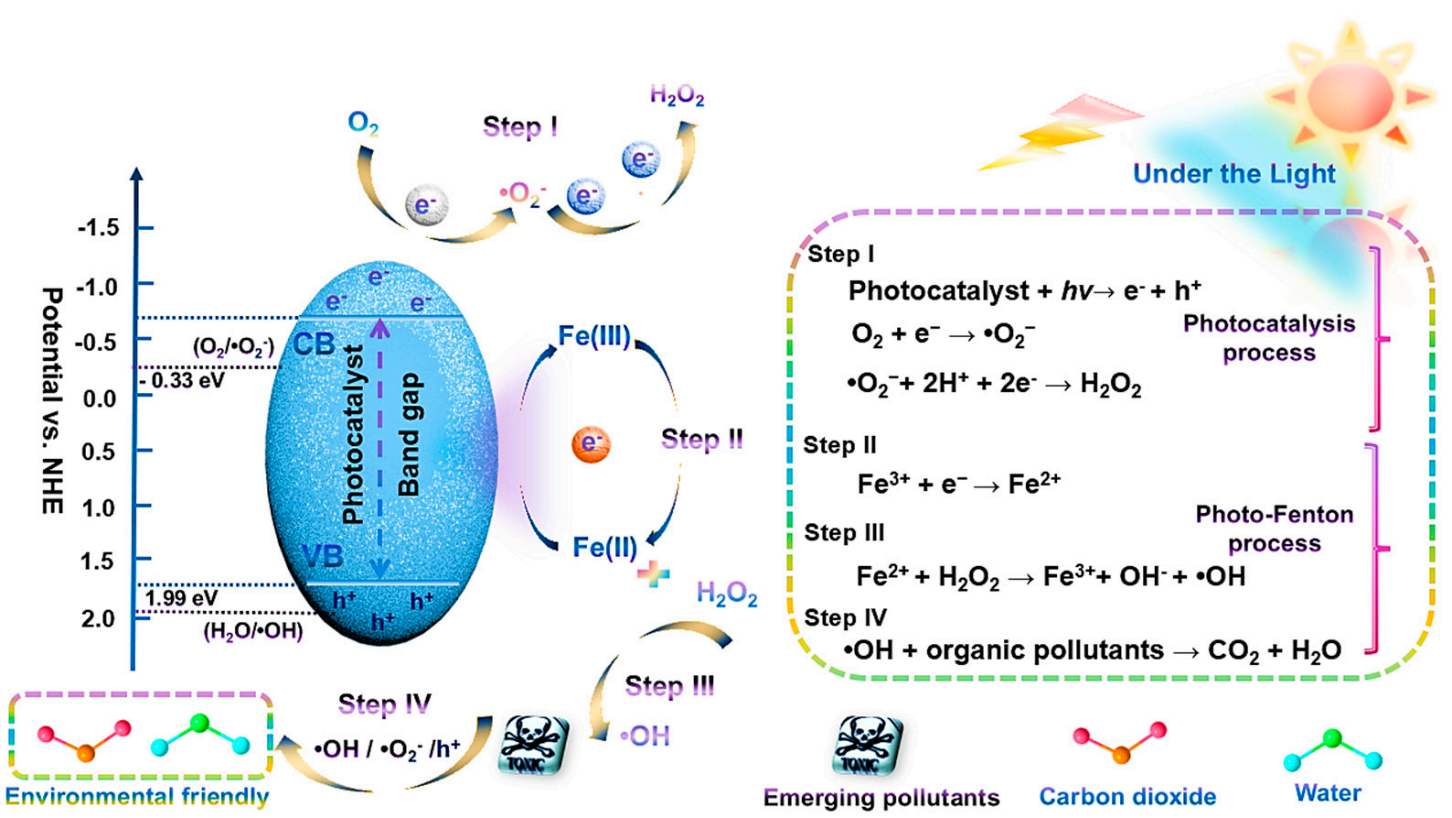
Schematic diagram of the mechanism of the photocatalytic self-Fenton system for the degradation of emerging pollutants.

The process of the degradation of emerging pollutants by the photocatalytic-self-Fenton system (Scheme 2) is mainly divided into two steps: (i) the photocatalyst can selectively and efficiently synthesize in situ H_2_O_2_ by solar-driven (i.e., photocatalytic process); (ii) the chain reaction between H_2_O_2_ and Fe^2+^ generates highly oxidized •OH, and then realizes efficient, green, and mineralization of various emerging pollutants (i.e., photo-Fenton process) [47].

### 2.1. Mechanisms of the H_2_O_2_ Production by Photocatalyst

A prerequisite for the efficient photocatalytic-self-Fenton degradation of emerging pollutants is the ability of the photocatalyst to produce a sufficient amount of H_2_O_2_. Photo-driven H_2_O_2_ production based on photocatalysts involves a series of complex and continuous photocatalytic reactions, which mainly consist of three consecutive reaction steps: (i) after the photocatalyst absorbs light energy greater than its band gap, the electrons (e^−^) are excited to migrate from the valence band (VB) to the conduction band (CB), leaving holes (h^+^) in the VB (Equation (1)); (ii) photo-excited electrons and holes migrate to the surface of the photocatalyst and participate in the surface redox reactions to produce reactive oxygen species (ROS); (iii) during this photocatalytic reaction, the carriers inevitably tend to recombine to form electron–hole pairs, while the surviving carriers migrate to the catalyst surface to participate in H_2_O oxidation and O_2_ reduction reactions (Equations (2) and (3)) and generate H_2_O_2_.

(1) $$\mathrm{photocatalyst}+\mathrm{hv}\to \mathrm{photocatalyst}\;\left({\;h}^{+}+e^{-}\right)$$

(2) $$e^{-}+O_{2}\to O_{2}^{-}$$

(3) $$O_{2}+2e^{-}+2H^{+}\to H_{2}O_{2}$$

In addition, since the research process of H_2_O_2_ production mainly focuses on the reduction of O_2_, it can also be achieved through the two-step single-e^−^ oxygen reduction reaction (Equations (4)–(6)) [48,49,50]. Specifically, during the ORR, the photo-induced electrons of the photocatalyst conduction band can react with O_2_ adsorbed on the surface to produce $O_{2}^{.-}$ (Equation (2)), while O_2_ can also react with H^+^ to produce H$O_{2}^{.-}$ (Equation (4)) as well as through the single-electron reduction process to produce HO_2_^−^ (Equation (5)), and finally, the reaction with H^+^ to produce H_2_O_2_ (Equation (6)). It is worth noting that although four-electron ORR is beneficial to O_2_ generation (Equation (7)), the selectivity for the formation of H_2_O_2_ from oxygen is diminished [51,52,53]. In addition, as the generation of $O_{2}^{-}$ needs more negative potential (−0.33 V) than the one-step double electron reaction (+0.68 V) and involves numerous unpredictable reactions, the two-step one-electron ORR reduces the H_2_O_2_ yield and selectivity [41,54]. In summary, the one-step double electron^−^ oxygen reduction pathway is more advantageous for the production of light-induced H_2_O_2_.

(4) $$O_{2}+H^{+}\to {\mathrm{HO}}_{2}^{-}$$

(5) $${\mathrm{HO}}_{2}^{.-}+e^{-}\to {\mathrm{HO}}_{2}^{-}$$

(6) $${\mathrm{HO}}_{2}^{-}+H^{+}\to H_{2}O_{2}$$

(7) $$O_{2}+4e^{-}+4H^{+}\to 2H_{2}O$$

### 2.2. Mechanisms of the Fe^2+^/H_2_O_2_-Mediated Fenton Reaction to Remove Emerging Pollutants

In 1894, Fenton technology was first proposed by the famous scientist Fenton for water treatment, mainly because ferrous ion (Fe^2+^) reacts with H_2_O_2_ to produce •OH with high oxidation capacity, which oxidizes and decomposes many refractory pollutants [55]. In addition, Haber et al. first studied the traditional homogeneous Fenton reaction between Fe^2+^ and H_2_O_2_ and formed an extraordinary complicated chain Fenton reaction, which is mainly divided into three chain steps: the initiation, propagation, and termination reaction [56]. In detail, these steps include (i) the production of •OH through oxidation reactions, at the same time, Fe^2+^ is oxidized to Fe^3+^ (Equation (8)); (ii) •OH with strong oxidation capacity and high electronegativity can react with organic compounds to form organic radical intermediates and hydroxylation products through the redox reaction and dehydrogenation reaction (Equation (9)) [47,57,58]; (iii) emerging pollutants react with •OH to form H_2_O andCO_2_ (Equation (10)).

(8) $${\mathrm{Fe}}^{3+}+e^{-}\to {\mathrm{Fe}}^{2+}$$

(9) $${\mathrm{Fe}}^{2+}+H_{2}O_{2}\to {\mathrm{Fe}}^{3+}+{\mathrm{OH}}^{-}+•\mathrm{OH}$$

(10) $$•\mathrm{OH}+\mathrm{emerging}\;\mathrm{pollutants}\to {\mathrm{CO}}_{2}+H_{2}O$$

However, it is inevitable that excess H_2_O_2_ may further react with the generated •OH to produce ${\mathrm{HO}}_{2}^{-}$, while Fe^3+^ can also react with H_2_O_2_ and then be reduced to Fe^2+^; H_2_O_2_ is oxidized to ${\mathrm{HO}}_{2}^{-}$. Therefore, the use of the initiation and propagation responses in the Fenton reaction system to avoid these unwanted termination reactions remains a considerable challenge.

### 2.3. The Influence Factors of H_2_O_2_ Generation

Efficient photocatalytic H_2_O_2_ production mainly depends on the suitable reaction conditions. First of all, suitable solvents (ethanol and isopropanol) provide a substantial role for photocatalytic H_2_O_2_ production as they can not only serve as electron donors to consume holes, but also promote the separation and migration of photo-generated carriers, so that more electrons can be used to reduce O_2_ [59,60]. In the next place, since the content of protons is closely related to the production of H_2_O_2_ based on the ORR pathway, in general, acidic conditions (pH < 7) are conducive to proton coupled electron transfer and promote H_2_O_2_ production, whereas alkaline conditions (pH > 7) may produce excess protons, leading to the further reaction of H_2_O_2_ with H_2_O [61,62,63]. At length, sufficient O_2_ is the key to ensure a high H_2_O_2_ yield, so the O_2_ consumed can be replenished continuously through the continuous supply of oxygen and four-electron water oxidation reactions during the reaction.

### 2.4. The Influence Factors of Fenton Reaction

The activity of the Fenton reaction greatly determines the mineralization efficiency of the pollutants, and the conventional homogeneous Fenton reaction is mainly affected by the following aspects: (i) pH value, as the activity of the Fenton reaction is limited by the pH value of the reaction solution because the Fenton reaction is conducive to the reaction of ferrous ions with H_2_O_2_ to form ·OH under acidic conditions (pH = 3–4) [64]; (ii) H_2_O_2_ concentration, as the concentration of H_2_O_2_ is critical to the activity of Fenton reaction, so a low concentration will inhibit the reaction rate, while a high concentration will not only lead to low utilization and precipitation, but Fe^2+^ will also be oxidized to Fe^3+^ at the beginning of the reaction, thus inhibiting the production of •OH [65,66]; (iii) Fe^2+^ concentration, as the reaction rate is slow when the concentration is too low, at high concentration, H_2_O_2_ will be reduced, and Fe^2+^ will be oxidized to Fe^3+^ [67,68].

## 3. Graphite Carbon Nitride (g-C_3_N_4_) Material

### 3.1. Synthetic Methods and Morphology of g-C_3_N_4_

Due to the small specific surface area of g-C_3_N_4_, the low utilization efficiency of visible light and the easy recombination of the photogenerated electrons and holes, researchers have improved the photocatalytic activity of g-C_3_N_4_ through different synthesis methods and morphological regulation [69,70,71].

As one of the most commonly used synthesis methods of g-C_3_N_4_, thermal polymerization, which has the advantages of simple operation, safety, and easy control, produces the triazine ring structure g-C_3_N_4_ by the pyrolysis polymerization of nitrogen-containing organic compounds such as melamine, urea, and thiourea at high temperature [72,73,74,75]. For example, Li et al. used melamine as a precursor to obtain g-C_3_N_4_ with curled edges through two-step thermal polymerization [76]. However, it is worth noting that compared with traditional low-dimensional materials, g-C_3_N_4_ obtained by the thermal polymerization of precursors is mostly of a block or lamellar structure, which is unfavorable to the photogenerated carrier separation and transport, thus limiting its photocatalytic activity. However, the solvothermal method can, by the thermal polymerization of organic precursors, form g-C_3_N_4_ with a nanoparticle or nanosheet structure under high temperature solvent conditions to solve the limitations of thermal polymerization method [77,78]. For example, Zhou et al. successfully prepared g-C_3_N_4_ metal-free composite (CNQDs/CN) modified with g-C_3_N_4_ quantum dots (CNQDs) by a simple solvothermal method, which significantly improved the photogenerated charge separation and migration rate of g-C_3_N_4_, thus showing excellent photocatalytic degradation activity [79].

Furthermore, the porous g-C_3_N_4_ with different nanostructures and morphologies can be obtained by depositing organic precursors in the template (silica gel, alumina) channels and conducting pyrolysis or chemical transformation [80,81]. Goettmann et al. used cyanamide as the precursor and SiO_2_ nanoparticles as the template; after heat treatment and condensation, g-C_3_N_4_ with high specific surface area and pore structure was obtained, which significantly increased its photocatalytic activity and light absorption capacity [82]. In addition to the above commonly used synthesis methods, there are some other methods such as microwave assisted synthesis, the sol–gel method, electrochemical synthesis, etc. that can achieve accurate control of the morphology and structure of g-C_3_N_4_ by regulating the reaction conditions, etc., thus providing diversified options for its application in the field of photocatalysis [83,84].

### 3.2. Band Structure of g-C_3_N_4_

**Scheme 3:**
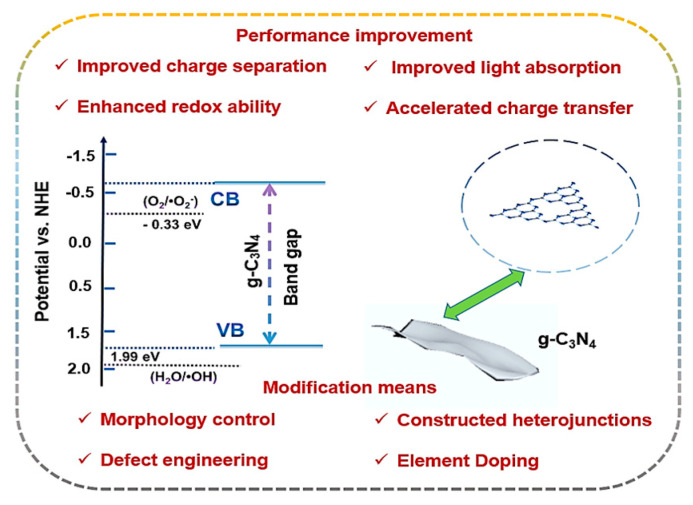
Schematic representation of the modification of the photocatalytic self-Fenton system based on the g-C_3_N_4_ photocatalyst.

In addition, graphite phase carbon nitride (g-C_3_N_4_), as a kind of carbide with a unique 2D layered structure, with easy to obtain raw materials, a medium band gap (2.70 eV), excellent chemical/thermal stability, and can absorb visible light at about 450–460 nm, is considered to be the most promising non-metallic visible light catalyst after TiO_2_ [85,86,87,88]. In particular, CN has the highest nitrogen content among known 2D carbon materials and the unique sp^2^-hybridization of C and N has been used to construct π-conjugated structures [89,90,91,92]. In addition, from the perspective of band position, the CB position of g-C_3_N_4_ (−1.27 V vs. NHE) is more negative than the standard potential ($O_{2}^{-}$: −0.33eV vs, NHE) of O_2_/$O_{2}^{-}$, so the electrons on the conduction band (CB) of g-C_3_N_4_ can effectively reduce O_2_ to $O_{2}^{-}$, which is more conducive to the generation of H_2_O_2_ in the photocatalytic self-Fenton reaction process through the double-electron oxygen reduction path. More importantly, the low valence band (VB) (1.4 eV vs. NHE) of g-C_3_N_4_ effectively prevents the oxidation of H_2_O_2_ during light irradiation [46,93,94,95]. Therefore, g-C_3_N_4_ is considered as a promising candidate for the in situ generation of H_2_O_2_. Unfortunately, some inherent scientific problems including the easy recombination of electron–hole pairs, small specific surface area, and low utilization of visible light, limit the practical application of original g-C_3_N_4_ in the actual photocatalytic self-Fenton system [96,97,98,99]. In order to solve the above limitations, as shown in Table 1, scientists have carried out various physical and chemical modifications of g-C_3_N_4_ (Scheme 3) from the aspects of morphological control, defect engineering, element doping, heterojunction construction, etc., to enhance the visible light capture, carrier migration, and separation rate to further improve the photocatalytic self-Fenton degradation activity of g-C_3_N_4_ [100,101,102,103,104]. Furthermore, we integrated the full text abbreviations into Table 2.

## 4. Various Modification Strategies for Photocatalytic Self-Fenton Based on g-C_3_N_4_

### 4.1. Morphology Control

The regulation of the morphology of the photocatalyst can improve the specific surface area and expose more reactive sites, thus affecting the photocatalytic activity to a certain extent [126,127,128]. Unfortunately, the practical application of the original g-C_3_N_4_ is limited due to the small specific surface area, the high photo-generated carrier recombination rate, and weak van der Waals force interaction between layers [129,130,131]. Currently, the morphological adjustment work of g-C_3_N_4_ is mainly focused on high-dimensional porous engineering and low-dimensional nanostructure control including templating, supramolecular preorganization, and the exfoliation method (e.g., liquid phase ultrasound [132], chemical oxidation [133], and gas template stripping [134], etc.), successfully realizing the transformation from the bulk phase structure to nanosheets. For instance, Cui’s group prepared mesoporous CN (mpg-CN) nanosheets with a specific surface area of 180 m^2^g^−1^ using 12 nm silica particles as a template through the silicon hard template method [135]. Shortly after, Xu et al. used the thermal oxidation etch stripping approach to fabricate a two-dimensional (2D) graphene-like carbon nitride-like structure (GA-C_3_N_4_) with a specific surface area of 30.1 m^2^g^−1^ [136]. It is worth noting that the construction of porous and hierarchical 2D g-C_3_N_4_ can not only enhance the light absorption, but also promote photo-induced charge separation and migration by shortening the transmission distance, which is conducive to the photocatalytic in situ production of H_2_O_2_ as an intermediate to enable the production of •OH for the degradation of visible light-driven pollutants [129].

**Figure 1:**
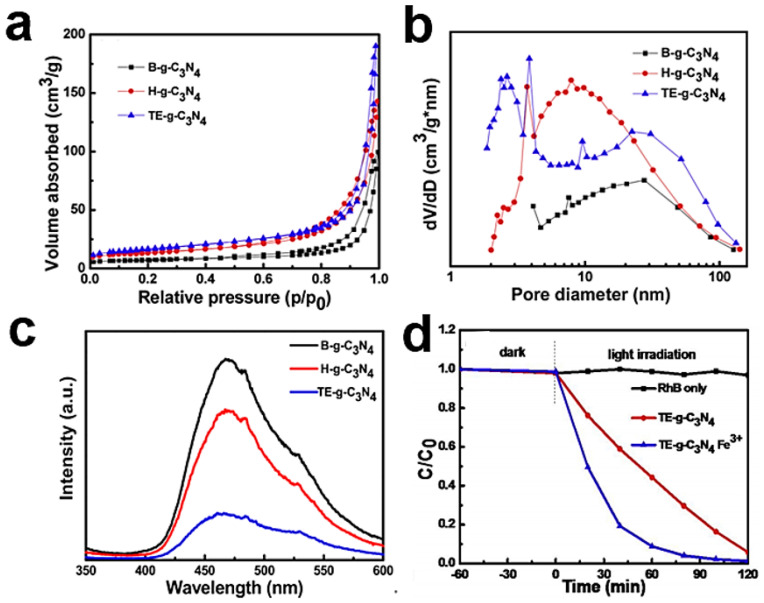
(**a**) Nitrogen adsorption–desorption isotherms and (**b**) Barrett–Joyner–Halenda (BJH) pore size distribution plots for the B-g-C_3_N_4_, H-g-C_3_N_4_, and TE-g-C_3_N_4_ samples, respectively. (**c**) Photoluminescence PL spectra of B-g-C_3_N_4_, H-g-C_3_N_4_, and TE-g-C_3_N_4_. (**d**) Removal efficiency of RhB using TE-g-C_3_N_4_ [107]. Copyright 2020, Elsevier.

The specific surface area (Figure 1a) and pore volume (Figure 1b) of TE-g-C_3_N_4_ nanosheets prepared by Li et al. by the thermal exfoliation method have been greatly improved compared with the bulk g-C_3_N_4_ (B-g-C_3_N_4_), providing more abundant reaction sites and light capturing ability [107]. In addition, due to the improvement in the TE-g-C_3_N_4_ photo-generated charge separation efficiency (Figure 1c), the in situ generation of H_2_O_2_ was promoted, and the efficient degradation of the organic pollutant rhodamine B (RhB) was quickly completed (Figure 1d) in the presence of Fe^3+^ (supplied by FeCl_3_) as the catalyst. To our knowledge, this work is the first to incorporate a practical application of photo-Fenton degradation pollutants without the external addition of H_2_O_2_.

**Figure 2:**
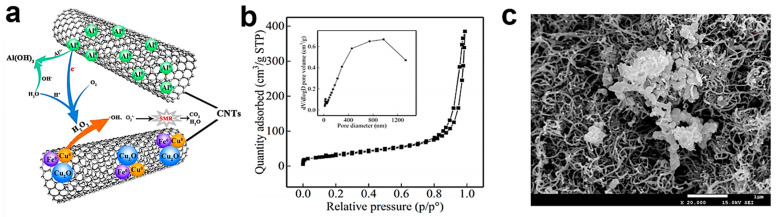
(**a**) The reaction mechanism of the Al^0^-CNTs/CNTs-Fe-Cu/O_2_ system. (**b**) N_2_ adsorption/desorption isotherm and the pore size distribution (the inset) of CNTs-Fe-Cu. (**c**) SEM image of the CNTs-Fe-Cu composite [108]. Copyright 2020, Elsevier.

In addition, the construction of three-dimensional (3D) structures also has a significant impact on the photocatalytic activity. Compared with the conventional 2D structure, the 3D structure has the advantages of larger specific surface area, a large number of active sites, and better light refraction, promoting photo-generated carrier separation and inhibiting carrier recombination [137]. Chen et al. used a novel catalyst (CNT-Fe-Cu) combined with Al^0^-carbon nanotubes (CNTs) to construct a photocatalytic self-Fenton system under visible light irradiation (Figure 2a) [108]. Among them, the Al^0^-CNTs composite, due to its 3D tubular structure, has a large specific surface area (Figure 2b), which promotes photo-generated electron separation and migration; H_2_O_2_ can be generated in situ under oxygen aeration, while the CNT-Fe-Cu composite can convert H_2_O_2_ into •OH and O_2_^−^ radicals. The deposition of Fe and Cu metals on the surface of CNTs improved the catalytic activity (Figure 2c), resulting in the efficient degradation of *sulfamerazine* (SMR) at neutral pH. Although morphology control is a very important means by which to improve the photocatalytic performance of materials, the degree of modification is still low, so other modification strategies are often combined to improve the photocatalytic reactivity. Dang et al. modified CN by combining morphology regulation and P doping using isopropyl alcohol as the hole scavenger, where the H_2_O_2_ generation rate under visible light was up to 1684 μmol h^−1^ g^−1^, which was 11.2 times the volume of pristine CN [138]. Due to the synergistic effect of the two modification methods, the change in morphology significantly enhances the specific surface area and light response, and the P element doping effectively regulates the band structure and promotes the separation of photoinduced carriers, thus enhancing the photocatalytic activity.

### 4.2. Defect Engineering

In recent years, introducing defects (such as oxygen vacancy [139], sulfur vacancy [140], carbon vacancy [141], and nitrogen vacancy [142]) with appropriate concentrations in photocatalysts has been proven to be an effective way to improve photocatalytic activity. On the one hand, the introduction of defects in g-C_3_N_4_ can adjust its band structure, activate oxygen molecule, narrow the band gap, and effectively broaden the visible light absorption range. On the other hand, the defect can act as the capture center of the photo-generated carrier, which can effectively reduce the recombination rate of photo-induced electron–hole pairs and improve the photocatalytic H_2_O_2_ generation [143]. In a typical case, Zhang et al. introduced double defect sites (-C≡N groups and N-vacancy) sequentially into g-C_3_N_4_ (Nv-C≡N-CN), forming an electron-rich structure that resulted in a more local charge density distribution to improve the visible light absorption and photo-generated carrier separation and migration [144]. In addition, the N-vacancy and -C≡N groups can activate O_2_ and promote the adsorption of H^+^, respectively, thus significantly improving the H_2_O_2_ generation. As a result, Nv-C≡N-CN reached the H_2_O_2_ generation rate of 3093 mmol g^−1^ h^−1^, which was 8.7 times stronger than the original CN (PCN) under visible light. Notably, the introduction of N defects can narrow the band gap of the photocatalyst and form a defect state, thereby enhancing light capture and accelerating the photo-generated carrier separation and migration [145].

**Figure 3:**
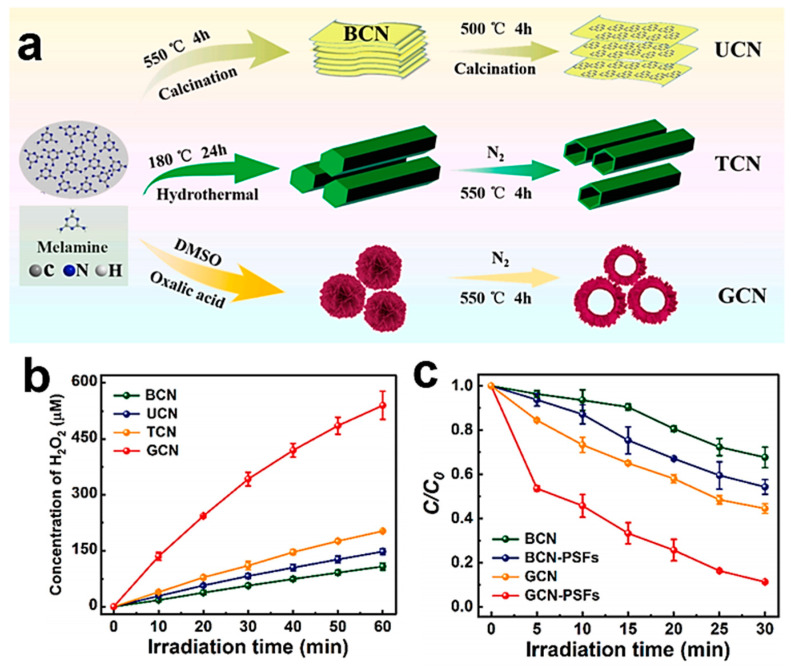
(**a**) Synthesis schematic diagram of g-C_3_N_4_ with four different morphologies (BCN, UCN, TCN, and GCN). (**b**) Photocatalytic H_2_O_2_ production of BCN, UCN, TCN, and GCN under visible light irradiation. (**c**) The photocatalytic decomposition curves of 2,4-DCP with BCN, BCN-PSFs, GCN, and GCN-PSFs under visible light irradiation. Reproduced with permission [114]. Copyright 2022, Elsevier.

Wu et al. constructed a photocatalytic self-Fenton system (GCN-PSFs) for the degradation of 2,4-dichlorophenol (2,4-DCP) by introducing carbon defects into garland g-C_3_N_4_ (Figure 3a) [114]. Notably, in the absence of a sacrificial agent, the introduction of carbon defects not only accelerated charge separation, but also improved the double-electron ORR, resulting in a H_2_O_2_ yield of 507.82 μM g^−1^ h^−1^ for garland g-C_3_N_4_ (GCN) (Figure 3b). Subsequently, compared with pure BCN and GCN, the photocatalytic degradation 2,4-DCP activities of BCN-PSFs and GCN-PSFs formed by adding Fe^2+^ to the photocatalytic degradation system could reach 45.8 and 88.8%, respectively (Figure 3c). The improvement in the photocatalytic degradation activity is mainly attributed to the following reasons: (i) the introduction of carbon defects accelerates the separation of photo-generated electron and holes, improving the adsorption capacity of O_2_ and the production of H_2_O_2_ by double-electron ORR; (ii) the stable production of H_2_O_2_ can effectively transform into •OH to degrade organic pollutants.

**Figure 4:**
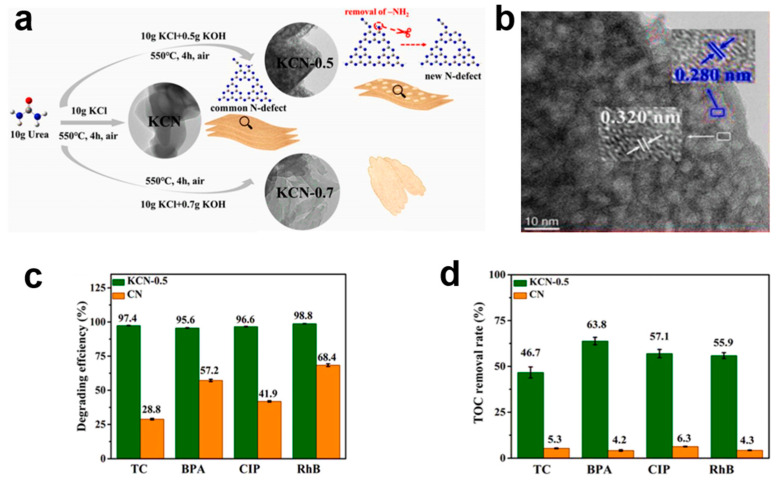
(**a**) Schematic diagram of the formation process of the as-prepared catalysts. (**b**) HR-TEM images of KCN-0.5. (**c**,**d**) Degrading efficiencies and the TOC removal rates of organic pollutants by KCN-0.5 and CN. Reproduced with permission [125]. Copyright 2023, Elsevier.

In addition, under suitable basicity, Yue et al. used the one-step thermal polymerization method of urea in KOH-KCl to construct a novel carbon nitride (CN) with abundant surface N defects and compressed π–π layer stacking for the photocatalytic self-Fenton removal of organic pollutants in water (Figure 4a) [125]. The high-resolution transmission electron microscopy (HR-TEM) images in Figure 4b display two different spacing lattice fringes, which are due to the stacking distances of different π–π layers at the surface and bulk phases, forming a double structure [146]. Due to the unique surface N defect that transfers photo-generated electrons to O_2_, KCN-0.5 exhibited an efficient in situ H_2_O_2_ production of 3.69 mM h^−1^ g^−1^ under λ = 420 nm and an aqueous solution containing IPA. As expected, KCN-0.5 exhibited excellent photocatalytic self-Fenton degradation and mineralized organic pollutants (tetracycline hydrochloride, bisphenol A, ciprofloxacin, rhodamine B) efficiency (Figure 4c,d). Therefore, the regulation of defects can promote the generation of H_2_O_2_ and further activate the H_2_O_2_ into ·OH (H_2_O_2_ + e^−^ → ·OH + OH^−^), so as to improve the photocatalytic self-Fenton degradation activity of emerging pollutants.

### 4.3. Constructed Heterojunctions

**Scheme 4:**
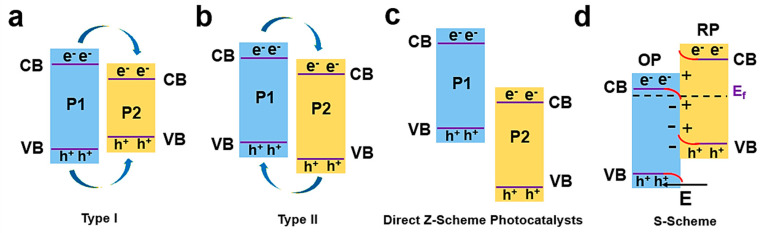
Schematic representation of the traditional (**a**) Type-I, (**b**) Type-II, (**c**) Z-scheme, and (**d**) S-scheme electron transfer paths.

Coupling g-C_3_N_4_ to other photocatalysts with a staggered band structure (including metals, metal oxides, inorganic semiconductors, conductive polymers, etc.) by synthetic means to form heterostructures is also an effective way to improve the separation and migration of photogenerated carriers and improve their photocatalytic activity [147,148,149]. Moreover, compared with other commonly used g-C_3_N_4_ structural modification methods, the establishment of heterogeneous structures has the advantage of being more controllable and facile [150]. Generally speaking, heterojunctions are divided into traditional heterojunctions (Type-I, II), Z-scheme heterojunctions, and emerging S-scheme heterojunctions (Scheme 4).

**Figure 5:**
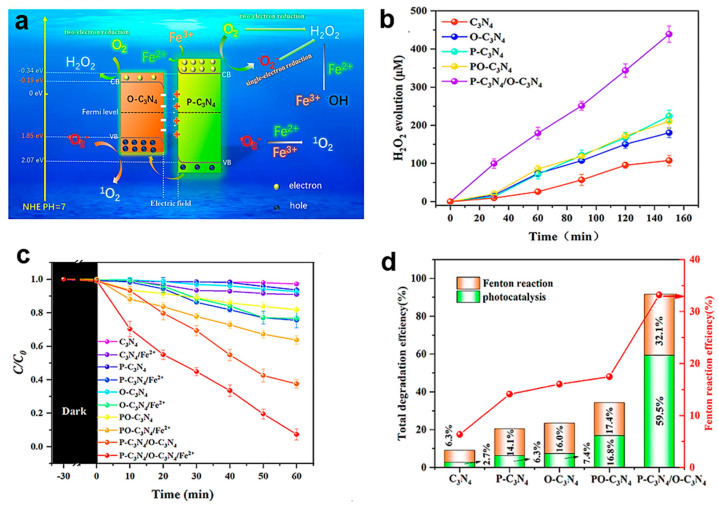
(**a**) The possible mechanism in the photocatalysis-self-Fenton reaction using the Type-I P-C_3_N_4_/O-C_3_N_4_ isotype heterojunction. (**b**) Photocatalytic H_2_O_2_ production in an air-saturated solution with EtOH as scavenger of h^+^ in the presence of various photocatalysts. (**c**) MTZ degradation efficiency in the photocatalytic process and photocatalysis-self-Fenton process using different photocatalysts. (**d**) The contribution of the photocatalytic process and Fenton reaction in the presence of various photocatalysts [112]. Copyright 2022, Elsevier.

Due to the difference in band structure between the phosphorus-doped C_3_N_4_ (P-C_3_N_4_) and oxygen-doped C_3_N_4_ (O-C_3_N_4_), Li et al. prepared a P-C_3_N_4_/O-C_3_N_4_ Type-I heterojunction as an efficient photocatalytic self-Fenton reaction catalyst (Figure 5a) [112]. From the transmission electron microscopy (TEM), it is evident that P-C_3_N_4_/O-C_3_N_4_ had been successfully synthesized. Under visible light irradiation, the formation of a Type-I heterojunction effectively promoted the photo-induced carrier separation with the help of an internal-electric-field, and the in situ generation rate of H_2_O_2_ was as high as 179 μM g^−1^ h^−1^ (Figure 5b). In the presence of Fe^2+^, the photocatalytic self-Fenton degradation of metronidazole (MTZ) by the P-C_3_N_4_/O-C_3_N_4_ Type-I heterojunction in Fenton within 60 min was 91.6%, which is 10.3 times that of pure C_3_N_4_ (Figure 5c,d). However, in the conventional Type-I heterojunction, since the semiconductor band structure is easily included, electrons and holes tend to migrate to the other semiconductor for recombination, which is not conducive to carrier separation.

**Figure 6:**
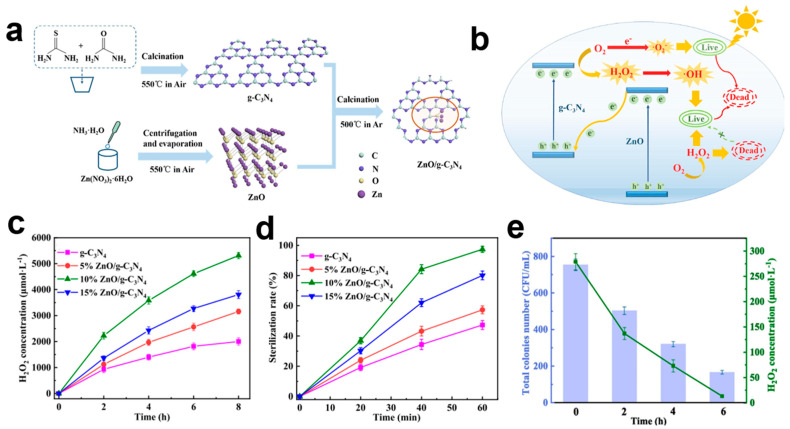
(**a**) Synthetic process of the ZnO/g-C_3_N_4_ heterojunction photocatalyst. (**b**) Pathway of H_2_O_2_ production and photocatalytic sterilization. (**c**) H_2_O_2_ productivity under simulated sunlight over various catalysts. (**d**) Sterilization efficiency for bacteria over different photocatalysts. (**e**) Residual sterilization performance after the photocatalytic process [110]. Copyright 2021, Elsevier.

Fortunately, for the Type-II heterojunction, due to the interleaving semiconductor band structure, electrons and holes are easily separated in space, thus reducing carrier recombination to further promote photocatalytic self-Fenton activity. Geng et al. prepared a novel ZnO/g-C_3_N_4_ Type-II heterojunction photocatalytic self-Fenton system by the thermal polycondensation process (Figure 6a) for the in situ sterilization in natural water under simulated sunlight [110]. By constructing a Type-II heterojunction, the response ability of the ZnO/g-C_3_N_4_ catalyst to sunlight and photoinduced charge separation was improved (Figure 6b), and the in situ H_2_O_2_ production and sterilization performance of the 10 wt% ZnO/g-C_3_N_4_ heterojunction catalyst within 60 min were 5312.45 μmol⋅L^−1^ and 97.4%, respectively (Figure 6c,d). It is worth mentioning that after the end of the disinfection process, the number of colonies and the concentration of hydrogen peroxide continued to decrease (Figure 6e), which may be due to the fact that there was still a certain concentration of hydrogen peroxide that could kill bacteria in the solution after the end of the photocatalysis. Unfortunately, due to the existence of electrostatic repulsion, Type-II heterojunctions still face the problem of poor redox capacity [151].

**Figure 7:**
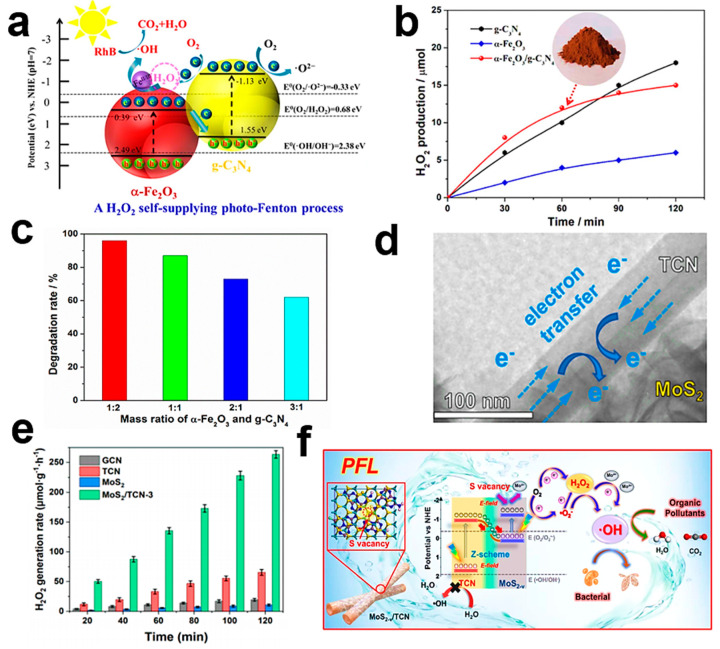
(**a**) Schematic illustration of the photo-generated charge transfer and degradation mechanism of organic pollutants in α-Fe_2_O_3_/g-C_3_N_4_. (**b**) The yields of H_2_O_2_ at different catalysts under simulated solar light illumination. (**c**) Effect of the Fe:C ratio on the degradation rate of RhB at 90 min [93]. Copyright 2020, Elsevier. (**d**) HRTEM image of MoS_2-v_/TCN-3. (**e**) The amount of in situ generation of H_2_O_2_ through different catalysts. (**f**) Performance enhancement mechanism of the PFL(MoS_2-v_/TCN-3) system [120]. Copyright 2023, Elsevier.

Based on the Z-scheme heterojunction simulated by natural plant photosynthesis, it could not only improve the photo-generated carrier separation efficiency, but also retained strong redox potential, thus overcoming the defects of Type-II heterojunction. Recently, Wang et al. prepared an efficient Z-scheme α-Fe_2_O_3_/g-C_3_N_4_ photocatalytic self-Fenton system (Figure 7a) by using α-Fe_2_O_3_ as the heterogeneous Fenton catalyst coupled with a g-C_3_N_4_ narrow band gap semiconductor [93]. The formation of a Z-scheme heterojunction not only promotes the separation of photogenerated electron–hole pairs, but also maintains the strong redox ability of electron–holes pairs in the g-C_3_N_4_ CB and α-Fe_2_O_3_ VB, which is conducive to the in situ formation of H_2_O_2_ (Figure 7b) and the efficient degradation of rhodamine B (RhB) and tetracycline hydrochloride (TC-H) (Figure 7c). Furthermore, Jiang et al. constructed the photocatalytic self-Fenton reaction system based on molybdenum disulfide with S-vacancy/carbon nitride nanotube (MoS_2-v_/TCN) composites (Figure 7f) for in situ H_2_O_2_ generation and the decomposition of micropollutants/bacteria over a wide pH range [120]. The unique tubular hollow structure of TCN provides more active sites, reduces the migration distance of photogenerated carriers, and promotes the close and tight contact between TCN and MoS_2-v_ at the interface (Figure 7d). Combined with density functional theory (DFT) calculation, the construction of Z-scheme heterojunction promotes the rapid separation of photo-induced e^−^ and h^+^, greatly reduces the energy barrier production of •OOH and •HO_2_^−^, and effectively improves the photocatalytic production of H_2_O_2_ (Figure 7e) and the degradation of organic pollutants by the MoS_2-v_/TCN heterojunction.

In summary, the construction of a Z-scheme heterojunction is considered to be an effective modification method, which can improve the photocatalytic activity. Nevertheless, the Z-scheme heterojunction also has some drawbacks including unpredictable side reactions, sensitivity to pH, and controversial charge transfer routes [152]. In recent years, S-scheme heterojunctions composed of oxidized and reduced photocatalysts with staggered band structures have shown broad prospects in the field of photocatalysis, which promote the separation of photo-generated electrons and holes due to their internal electric field, band bending, and electrostatic interactions [153]. For instance, Pham et al. constructed S-scheme heterojunction α-Fe_2_O_3_/g-C_3_N_4_ nanocomposites that could efficiently degrade cephalexin and amoxicillin under visible light [154]. Meanwhile, Wei et al. reported for the first time an S-scheme heterojunction Co_3_O_4_/Bi_2_MoO_6_@g-C_3_N_4_ hollow microsphere that effectively promoted the separation of photogenerated electrons and holes due to the formation of internal electric field, and had a degradation rate of levofloxacin (LVFX) up to 95.21% under visible light irradiation [155]. In addition, it is worth noting that the construction of an S-scheme heterojunction photocatalyst based on g-C3N4 is also conducive to the production of H_2_O_2_. Xia et al. synthesized an S- scheme Zn-TCPP/CN composite by grafting g-C_3_N_4_ with supramolecular zinc porphyrin (Zn-TCPP) using ethanol as a sacrificial agent, and showed a H_2_O_2_ production rate with 532.7 μmol/L within 90 min, which is 3.1 times and 9.0 times of pure CN and Zn-TCPP, respectively [156]. Therefore, the development of new S-scheme photocatalysts based on g-C_3_N_4_ may be a future direction to improve the activity of photocatalytic self-Fenton degradation.

### 4.4. Element Doping

Element doping refers to the method of introducing metal/non-metal elements into the semiconductor in a certain way. In general, the non-metallic elements doped into g-C_3_N_4_ will replace the C and N elements of g-C_3_N_4_ itself, and the metal elements will be embedded in the internal structure of g-C_3_N_4_, and the energy band structure of g-C_3_N_4_ will change significantly after the element doping, improving the ability to capture sunlight and conductivity [157,158,159,160]. Furthermore, element doping is also an effective strategy to enhance photocatalytic activity by regulating the optical electronic properties of H_2_O_2_ generation [161]. Hu et al. synthesized hollow copper (Cu) doped graphitic carbon nitride (g-C_3_N_4_) microspheres, where Cu^+^ is inserted into the interstitial site in the form of a coordination bond (Cu(I)-N) and acts as a chemisorption site to activate the O_2_ molecule, thus exhibiting excellent H_2_O_2_ production performance (4.8 mmol L^−1^) [162].

**Figure 8:**
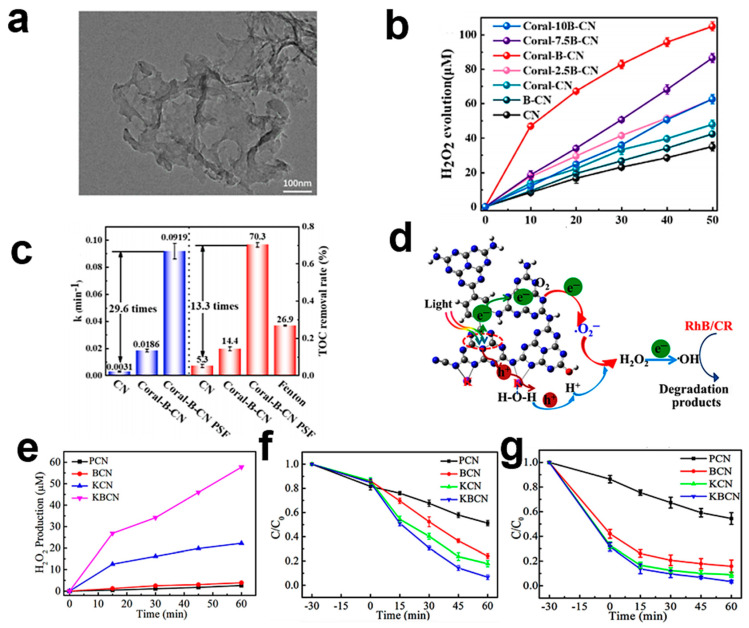
(**a**) TEM image of coral-B-CN. (**b**) H_2_O_2_ yield curves for different catalysts. (**c**) The k-value and TOC removal rate for different systems [122]. Copyright 2023, Elsevier. (**d**) Schematic of Fenton-like degradation of RhB and CR with in situ-generated H_2_O_2_ over KBCN. (**e**) Photocatalytic production of H_2_O_2_ in pure water. Results of the degradation efficiencies (**f**) for RhB and CR (**g**) over KBCN [117]. Copyright 2022, Elsevier.

Jing and co-workers fabricated a novel photocatalytic self-Fenton system based on a coral-like B-doped g-C_3_N_4_ (coral-B-CN) photocatalyst [122]. The transmission electron microscope (TEM) image in Figure 8a shows that coral-B-CN has a lamellar structure and an obvious pore distribution. Under simulated sunlight irradiation, as B heteroatom doping in coral-B-CN enhanced the molecular dipole, accelerated the charge separation rate, and enhanced the O_2_ adsorption, the H_2_O_2_ production activity of coral-B-CN (Figure 8b) reached 104.9 μM. As expected, the efficient carrier separation and migration rate of coral-B-CN accelerated the Fe^2+^/Fe^3+^ cycle, and the resulting H_2_O_2_ reacted with Fe^2+^ to form the ·OH radical, resulting in the efficient degradation and mineralization of 4-CP in the photocatalytic self-Fenton system (Figure 8c). Significantly, the co-doping of multiple elements can often achieve the effect of “1 + 1 > 2” by utilizing the synergy of each component [163]. For example, in the photocatalytic self-Fenton system, Chen et al. constructed benzene and K^+^ co-doped g-C_3_N_4_ (KBCN) by the thermal polymerization approach to degrade rhodamine B (RhB) and Congo red (CR) via in situ generated H_2_O_2_ under visible light irradiation (Figure 8d) [117]. Since benzene and K^+^ co-doping increased the photo-generated charge separation rate and promoted the in situ formation of H_2_O_2_ in KBCN, the H_2_O_2_ yield of KBCN was 57.5 mM in pure water after 60 min of visible irradiation (Figure 8e). As shown in Figure 8f,g, KBCN for the degradation of RhB and CR rate was 93.3% and 96.6%, respectively, after 60 min with H_2_O_2_-derived •OH radicals under the condition of natural pH and no Fe^2+^ ions added.

**Figure 9:**
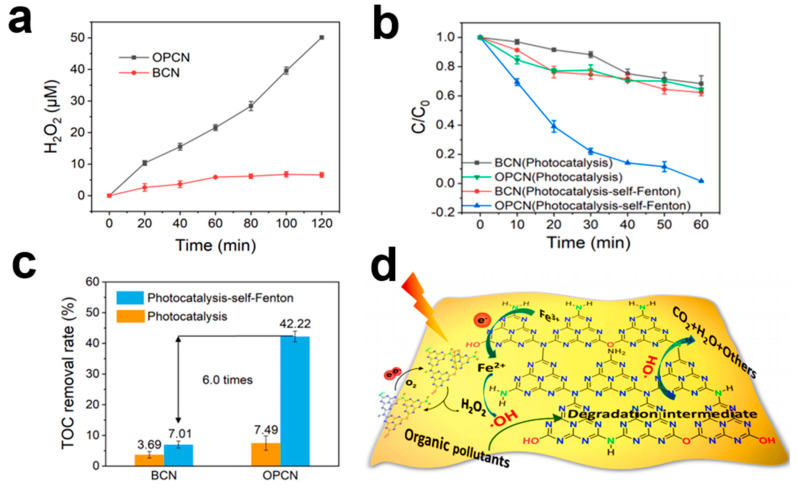
(**a**) The photocatalytic H_2_O_2_ generation curves of BCN and OPCN. (**b**) The 2,4-DCP degradation activity. (**c**) The TOC removal rate between photocatalysis-self-Fenton and photocatalysis based on OPCN and BCN. (**d**) Schematic illustration of the photocatalysis-self-Fenton system based on OPCN [24]. Copyright 2022, Elsevier.

It must also be mentioned that a single modification strategy may have some limitations in optimizing the photocatalytic activity, and it is an effective method to improve the self-Fenton performance of photocatalysis by integrating different modification strategies to maximize the synergistic effect. Theoretically, the combination of morphology regulation and element doping is a feasible method because the morphology regulation can solve the shortcomings of low specific surface area and slow carrier propagation efficiency, while element doping has great benefits in solving the light absorption capacity. For instance, Xu’s research group constructed a photocatalytic self-Fenton system based on oxygen-doped porous g-C_3_N_4_ nanosheets (OPCN) [24]. The well-developed porosity and high specific surface area of OPCN effectively promote the reduction of double electrons to O_2_, while the doping of oxygen containing groups enhances the water oxidation capacity, leading to the efficient generation of H_2_O_2_ (Figure 9a). Due to the efficient in situ formation of H_2_O_2_ by OPCN and the introduction of the FeCl_3_.6H_2_O reaction reagent containing Fe^3+^, the degradation rate of 2,4-DCP (Figure 9b) is 11.5 and 9.9 times higher than that of the g-C_3_N_4_ photocatalytic and Fenton system, while the mineralization rate (Figure 9c) is 11.4 and 4.2 times higher than that of g-C_3_N_4_ photocatalytic system, respectively. To sum up, the morphology of the porous nanosheets and the introduction of oxygen-containing groups promote the carrier transfer efficiency, strengthen the oxidation capacity of water, and lead to the highly selective generation of H_2_O_2_ on the surface of OPCN under visible light. At the same time, some electrons reduce Fe^3+^ to Fe^2+^, react with H_2_O_2_ to generate ·OH radicals, and degrade organic pollutants into water and CO_2_ (Figure 9d).

### 4.5. Others

#### 4.5.1. Heterojunction of g-C_3_N_4_ with Other Materials

The heterostructures formed between g-C_3_N_4_ and other materials (such as MOFs, metal oxides, single atom catalysts, etc.) can further enhance the photocatalytic reaction activity by improving the light absorption, optimizing the interfacial transport path between photogenerated carriers and enhancing the interfacial reaction characteristics. MOFs (metal–organic frameworks), as new porous nanomaterials with high porosity and low density that are constructed by metal ions and organic ligands, are widely favored in the field of photocatalysis [164,165,166,167]. For example, V. Muelas-Ramos et al. synthesized a g-C_3_N_4_/NH_2_-MIL-125 composite material where the presence of MOF (NH_2_-MIL-125) not only inhibited the recombination rate of the g-C_3_N_4_ photogenerated electron–hole pairs, but also increased the g-C_3_N_4_ specific surface area and pore volume, demonstrating an excellent photocatalytic degradation diclofenac rate [164].

However, compared with the conventional MOF materials, since metal oxides (e.g., TiO_2_, WO_3_, ZnO, etc.) have the advantages of stable structure, good biocompatibility, and convenient preparation, they are also widely used to form heterojunctions with g-C_3_N_4_ to improve the optical absorption of composite materials, photocarrier separation, and the adsorption activity of reactants [168,169]. Wang et al. synthesized in situ a compact and uniform core-shell TiO_2_@g-C_3_N_4_ quantum heterojunction using urea and surface titanium dioxide as precursors [170]. Due to the unique structural advantages of the g-C_3_N_4_ shell and the compact and uniform contact interface with TiO_2_, it promoted photo-generated electron transfer and ·OH surface adsorption, thus significantly improving the degradation activity of photocatalytic tetracycline.

Moreover, it is worth noting that single-atom catalysts are widely used in the field of photocatalysis because of their advantages such as atomic-level active sites, high catalytic performance, low metal consumption, and stable structure [171]. Therefore, the heterojunctions formed by the hybridization of a single atomic catalyst and g-C_3_N_4_ creates new opportunities for improving the photocatalytic degradation performance. For instance, Wang et al. synthesized single-atom dispersed Ag modified mesoporous graphite carbon nitride (Ag/mpg-C_3_N_4_) by the co-condensation method [172]. The introduction of single-atom Ag enhanced the absorption and utilization of light and the Ag/mpg-C_3_N_4_ photocatalyst could degradation 100% bisphenol A (BPA) and mineralization of 80% TOC within 60 min.

#### 4.5.2. g-C_3_N_4_-Based Magnetic Photocatalysts

In addition, although g-C_3_N_4_ has the advantages of high thermodynamic and chemical stability and condonable energy band structure, it still suffers from the disadvantages of limited light absorption, low charge separation efficiency, and difficulty in recycling, which limit the photocatalytic performance of the material in practical applications [173]. Based on the advantages of g-C_3_N_4_-based magnetic photocatalysts such as excellent optical properties, thermal stability, and easy recycling, they have good prospects for the photocatalytic self-Fenton system. In 2021, Xiong et al. synthesized g-C_3_N_4_/diatomaceous earth/Fe_3_O_4_ composites by electrostatic self-assembly and added H_2_O_2_ to the system for the efficient degradation of rhodamine by the photocatalytic self-Fenton [174]. The incorporation of diatomaceous earth and magnetic material (Fe_3_O_4_) narrowed the band gap of the material, increased the light utilization rate, and improved the degradation ability of the g-C_3_N_4_-based photocatalyst. In addition, the composites had good recyclability. In 2022, Wang et al. synthesized magnetic Fe_3_O_4_/CeO_2_/g-C_3_N_4_ composites used for the efficient photocatalytic degradation of tetracycline hydrochloride (TCH) by the hydrothermal method [175]. The optimal material could remove 96.63% of TCH after 180 min of reaction. In addition, the composite had a lower leaching rate of Fe ions. Therefore, the g-C_3_N_4_-based magnetic photocatalyst can efficiently utilize photo-Fenton properties through the synergistic effect of semiconductors and magnetic materials as well as reduce environmental hazards by recycling materials.

## 5. Conclusions and Prospects

To conclude, this review summarizes the practical applications of g-C_3_N_4_-based photocatalysts in the photocatalytic self-Fenton system based on the existing literature and discusses in detail the mechanisms of photocatalytic self-Fenton in the degradation of emerging pollutants and the key factors affecting the degradation activity. In addition, according to the inherent defects of g-C_3_N_4_-based photocatalysts such as low light absorption utilization, few active sites, photogenerated electron–hole recombination, etc., different modification strategies to improve the performance of photocatalytic self-Fenton degradation were discussed in detail including morphological modulation, construction heterojunctions, defect engineering, and element doping strategies. Although the existing research on g-C_3_N_4_-based photocatalytic self-Fenton systems has made considerable progress from the perspective of adding additional Fe ions, the pH application range of the reaction system is narrow (pH ≤ 3), and Fe^3+^ is difficult to separate and recycle after the reaction, which cannot meet the actual needs of an efficient, green, and safe deep treatment technology that can be applied to the degradation and mineralization of emerging pollutants in the environment. In order to better improve the activity of a photocatalytic self-Fenton system, some insights and prospects are provided, as follows:

(1) In the photocatalytic self-Fenton process, the catalyst generates H_2_O_2_ by external interaction with Fe^2+^ in the photocatalytic phase, generating active species such as •OH by in situ activation, and promotes Fe^2+^/Fe^3+^ cycling for the efficient degradation of pollutants. However, most self-Fenton systems have problems such as metal leaching or agglomeration and difficult separation and recovery of Fe^3+^ after the reaction, leading to low material utilization and environmental pollution, so it is important to design and synthesize environmentally stable catalysts. Secondary contamination is avoided by constructing metal atom dispersion to hinder the leaching of iron atoms as well as the rational use of iron materials such as adding spinel ferrite materials to the system to form heterojunctions with the catalyst. The preparation of catalysts that are easy to synthesize, stable, easy to separate, and prevent metal ion leaching plays an important role in improving the performance of photocatalytic self-Fenton systems.
(2) As a photocatalytic system, the photocatalytic self-Fenton system is based on three basic processes of photocatalysis, whose photogenerated carrier separation and migration efficiency and photo-response range affect its activity. Currently, most g-C_3_N_4_-based photocatalysts are limited to the visible light region, and the near-infrared light, which accounts for 50% of the solar spectrum, is not effectively utilized, resulting in low overall light utilization of the catalyst. Although coupling with semiconductors and heteroatom doping can adjust the band gap to improve light absorption, the improvement in the light absorption range is limited based on the inherent nature of semiconductors. Therefore, it is a good strategy to achieve a broad-spectrum photo-response range by combining with near-infrared response materials containing metal elements such as Ag and Cu to generate a local plasmon resonance effect or up-conversion process.
(3) In addition to the catalyst properties themselves, external factors such as the pH of the reaction system and reaction solvent have significant effects on the catalyst activity. The conventional Fenton and Fenton-like reaction systems mainly generate active species such as hydroxyl groups via H_2_O_2_ and Fe^2+^ under acidic conditions, which limit the scope of catalyst applications, thus, it is important to design and synthesize catalysts with a wide pH response. Strategies such as the formation of iron complexes and the use of Fenton-like systems with a wide pH range can be used to prepare photocatalytic self-Fenton systems for the efficient degradation of pollutants.
(4) Sacrificial agents play an important role in the photocatalytic Fenton system, acting as electron acceptors or donors, trapping photogenerated electrons or holes and promoting photogenerated carrier separation efficiency. However, the widespread use of sacrificial agents lacks economic benefits, so the reaction performance can be improved by adding some functional small molecules that are not sacrificial agents. For example, when ethylenediaminetetraacetic acid (EDTA) is added to the reaction system, the electron-rich carboxyl groups in EDTA form hydrogen bonds with O_2_, which increases the electron density and bond length of O_2_ molecules, thus facilitating O_2_ activation and significantly increasing the H_2_O_2_ yield of the reaction system. In addition, functional small molecules such as oxalic acid and furfuryl alcohol were applied in the photocatalytic H_2_O_2_ production system, which significantly improved the H_2_O_2_ yield. Therefore, it is a direction worth exploring to effectively utilize the synergy between functional molecules and catalysts to improve the H_2_O_2_ yield and thus promote the subsequent Fenton reaction for the efficient degradation of pollutants.

## Figures and Tables

**Table 1:** Summary of the recent classification of pollutants degraded by the photocatalytic self-Fenton system based on g-C_3_N_4_. (N/A is not available).

Year	Photocatalysts	Light Source	Iron Ion Source	Sacrificial Agent	Pollutants	H_2_O_2_ Yield	Pollutant Removal (%)	TOC Removal (%)	Ref.
2020	P-g-C_3_N_4_	Vis	FeSO_4_·7H_2_O	-	2,4-DCP	23.5 μM h^−1^	91	42.7	[46]
	Ti^3+^-TiO_2_-C_3_N_4_por	Vis	-	-	MO	6.32 mM h^−1^	97.9	89	[105]
	M101-Ux	Vis	MIL101	-	2,6-DCP/2,4,5-TCP	23 μM h^−1^	98.7/97.3	16/14	[106]
	TE-g-C_3_N_4_	Vis	FeCl_3_	-	RhB	N/A	~100	-	[107]
	α-Fe_2_O_3_/g-C_3_N_4_	Vis	α-Fe_2_O_3_	IPA	RhB/TC	7.5 μM h^−1^	96/95	92/86	[93]
	Al^0^-CNTs/CNTs-Fe-Cu	Vis	FeSO_4_ 7H_2_O	-	SMR	N/A	85	60	[108]
	Metal-free GCN	Vis	Fe(II)	Isopropyl alcohol	GA	N/A	~93	88.8	[109]
2021	ZnO/g-C_3_N_4_	Vis	FeSO_4_	IPA	Bactericidal	5312.45 μM L^−1^	97.4	-	[110]
	SCNO@CdS	Vis	FeCl_2_·4H_2_O	IPA	RhB	79,971.0 μM g^−1^ h^−1^	~83	-	[111]
	P-C_3_N_4_/O-C_3_N_4_	Vis	FeSO_4_ 7H_2_O	IPA	MTZ	179 μM h^−1^	91.6	-	[112]
	g-C_3_N_4_/PDI	Vis	FeSO_4_ 7H_2_O	-	Bactericidal	112.95 μM h^−1^	~100	-	[113]
2022	GCN	Vis	FeSO_4_ 7H_2_O	IPA	2,4-DCP	21.59 mM h^−1^ g^−1^	88.8	-	[114]
	UPCN	Vis	FeCl_3_·6H_2_O	IPA	OTC	23.91 μM L^−1^	86.23	48..6	[115]
	Cg-C_3_N_4_	Vis	FeSO_4_ 7H_2_O	IPA	4-CP	217.26 μM g^−1^ h^−1^	~98	59.64	[116]
	KBCN	Vis	-	-	RhB/CR	57.7 μM h^−1^	93.3/96.6	-	[117]
	OPCN	Vis	FeCl_3_·6H_2_O	IPA	2,4-DCP	25.06 μM h^−1^	93	42.22	[24]
	CUCN	Vis	FeSO_4_ 7H_2_O	-	RhB	14.81 μM h^−1^	up to 100	63.77	[118]
	TFMS-CP	Vis	FeCl_3_	IPA	PH/HQ/MO	1.02 μM h^−1^	92.6/84.6/88.2	-	[119]
2023	MoS_2-v_/TCN	Vis	-	IPA	RhB	1879 μM g^−1^ h^−1^	98.5	-	[120]
	PC@PL	Vis	LaFeO_3_	-	MB	7.86 μM L^−1^	97	-	[121]
	Coral-B-CN	Vis	FeSO_4_ 7H_2_O	-	4-CP	314.55 μM g^−1^ h^−1^	99.6	70.3	[122]
	Cv-PCNNS	Vis	FeSO_4_ 7H_2_O	IPA	MTZ	984.8 μM L^−1^ h^−1^	90.7	62	[123]
	CPBA-CN	Vis	FeCl_2_·4H_2_O	-	4-CP	156.4 μM h^−1^	~97	74.6	[124]
	KCN	Vis	-	IPA	BPA	1.76 mM h^−1^	95.6	63.8	[125]

**Table 2:** Summary of the full text abbreviations.

Abbreviated Pre-Name	Abbreviated Name
Hydrogen peroxide	H_2_O_2_
Reactive oxygen species	ROS
Superoxide anion radical	$O_{2}^{-}$
Singlet oxygen	^1^O_2_
Graphite carbon nitride	g-C_3_N_4_
Oxygen reduction reaction	ORR
Advanced oxidation processes	AOPs
Hydroxyl radicals	∙OH
Valence band	VB
Conduction band	CB
2,4-dichlorophenol	2,4-DCP
Methyl Orange	MO
2,4,5-Trichlorophenol	2,4,5-TCP
Rhodamine B	RhB
Tetracycline	TC
2,6-dichlorophen	2,6-DCP
Sulfamerazine	SMR
Gallic acid	GA
Metronidazole	MTZ
4-Chlorophenol	4-CP
Phenol	PH
Hydroquinone	HQ
Oxytetracycline	OTC
Methylene Blue	MB
Metronidazole	MTZ
